# Andrographolide promotes hippocampal neurogenesis and spatial memory in the APPswe/PS1ΔE9 mouse model of Alzheimer’s disease

**DOI:** 10.1038/s41598-021-01977-x

**Published:** 2021-11-25

**Authors:** Sebastian B. Arredondo, Daniel T. Reyes, Andrea Herrera-Soto, Muriel D. Mardones, Nibaldo C. Inestrosa, Lorena Varela-Nallar

**Affiliations:** 1 Institute of Biomedical Sciences, Faculty of Medicine and Faculty of Life Sciences, Universidad Andres Bello, Echaurren 183, 8370071 Santiago, Chile; 2 Centro de Envejecimiento y Regeneración (CARE UC), Facultad de Ciencias Biológicas, Pontificia Universidad Católica de Chile, Av. Bernardo O´Higgins 340, P.O. Box 340-D, Santiago, Chile; 3 Centro de Excelencia en Biomedicina de Magallanes (CEBIMA), Universidad de Magallanes, Punta Arenas, Chile

**Keywords:** Adult neurogenesis, Alzheimer's disease, Spatial memory

## Abstract

In Alzheimer´s disease (AD) there is a reduction in hippocampal neurogenesis that has been associated to cognitive deficits. Previously we showed that Andrographolide (ANDRO), the main bioactive component of *Andrographis paniculate*, induces proliferation in the hippocampus of the APPswe/PSEN1ΔE9 (APP/PS1) mouse model of AD as assessed by staining with the mitotic marker Ki67. Here, we further characterized the effect of ANDRO on hippocampal neurogenesis in APP/PS1 mice and evaluated the contribution of this process to the cognitive effect of ANDRO. Treatment of 8-month-old APP/PS1 mice with ANDRO for 4 weeks increased proliferation in the dentate gyrus as evaluated by BrdU incorporation. Although ANDRO had no effect on neuronal differentiation of newborn cells, it strongly increased neural progenitors, neuroblasts and newborn immature neurons, cell populations that were decreased in APP/PS1 mice compared to age-matched wild-type mice. ANDRO had no effect on migration or in total dendritic length, arborization and orientation of immature neurons, suggesting no effects on early morphological development of newborn neurons. Finally, ANDRO treatment improved the performance of APP/PS1 mice in the object location memory task. This effect was not completely prevented by co-treatment with the anti-mitotic drug TMZ, suggesting that other effects of ANDRO in addition to the increase in neurogenesis might underlie the observed cognitive improvement. Altogether, our data indicate that in APP/PS1 mice ANDRO stimulates neurogenesis in the hippocampus by inducing proliferation of neural precursor cells and improves spatial memory performance.

## Introduction

Alzheimer’s disease (AD) is the most common cause of dementia with an estimated prevalence of 30 million people worldwide^1^. AD is a neurodegenerative disorder characterized by a progressive memory loss, impaired cognitive functions, massive neuronal loss and synaptic dysfunction^2^. Neuropathological hallmarks of AD include extracellular amyloid plaques formed by deposition of amyloid β peptide (Aβ) generated from amyloid precursor protein (APP) processing, and intracellular neurofibrillary tangles mainly composed by hyperphosphorylated tau protein^2,3^. In addition to neuronal loss, a decline in hippocampal neurogenesis was observed in AD patients^4–6^, which has also been evidenced in different animal models of the disease^7–10^. Interestingly, in AD mouse models impaired neurogenesis precedes Aβ plaque and neurofibrillary tangles formation^8,9,11,12^. This indicates that the impairment in neurogenesis is an early event in the disease progression, which has also been suggested in AD patients, where the decline in neurogenesis was observed even in individuals at early stages of the disease^4^.

In the adult hippocampus, new neurons are generated from neural stem cells (NSCs) located at the subgranular zone (SGZ) in the dentate gyrus. These cells proliferate giving rise to neural progenitor cells (NPCs) that differentiate into neuroblasts that develop and mature into granule neurons that became integrated into the hippocampal circuitry^13–15^. Adult-born neurons contribute to hippocampal plasticity^16–19^ and hippocampal-dependent tasks such as pattern separation, cognitive flexibility, and spatial learning and memory^20–22^. In humans, it was suggested a possible association between the extent of neurogenesis and cognitive status^5^. Therefore, the stimulation of neurogenesis has emerged as a possible therapeutic strategy in conditions affecting cognition.

Previously, we determined that Andrographolide (ANDRO), the main bioactive component of the medicinal plant *Andrographis paniculata*^23^, induced hippocampal neurogenesis in adult mouse brain^24^. ANDRO is a labdane diterpenoid lactone that has multiple therapeutic uses. It has shown protective effects in animal models of cerebral ischemia^25^, traumatic brain injury^26,27^, and neurodegenerative diseases^28–32^. ANDRO treatment reduced cognitive impairment in APPswe/PS1ΔE9 and J20 transgenic mouse models of AD^30–32^, as well as in *Octodon degus* used as a natural model of AD^33^. In addition, we determined that ANDRO induced cell proliferation in the hippocampus of APPswe/PS1ΔE9 mice^24^. Here we further characterized the effect of ANDRO treatment on hippocampal neurogenesis in APPswe/PS1ΔE9 mice and evaluated the potential contribution of neurogenesis in the effect of ANDRO on spatial memory improvement.

## Results

### ANDRO increases cell proliferation in the dentate gyrus of APPswe/PSEN1ΔE9 mice

**Figure 1:**
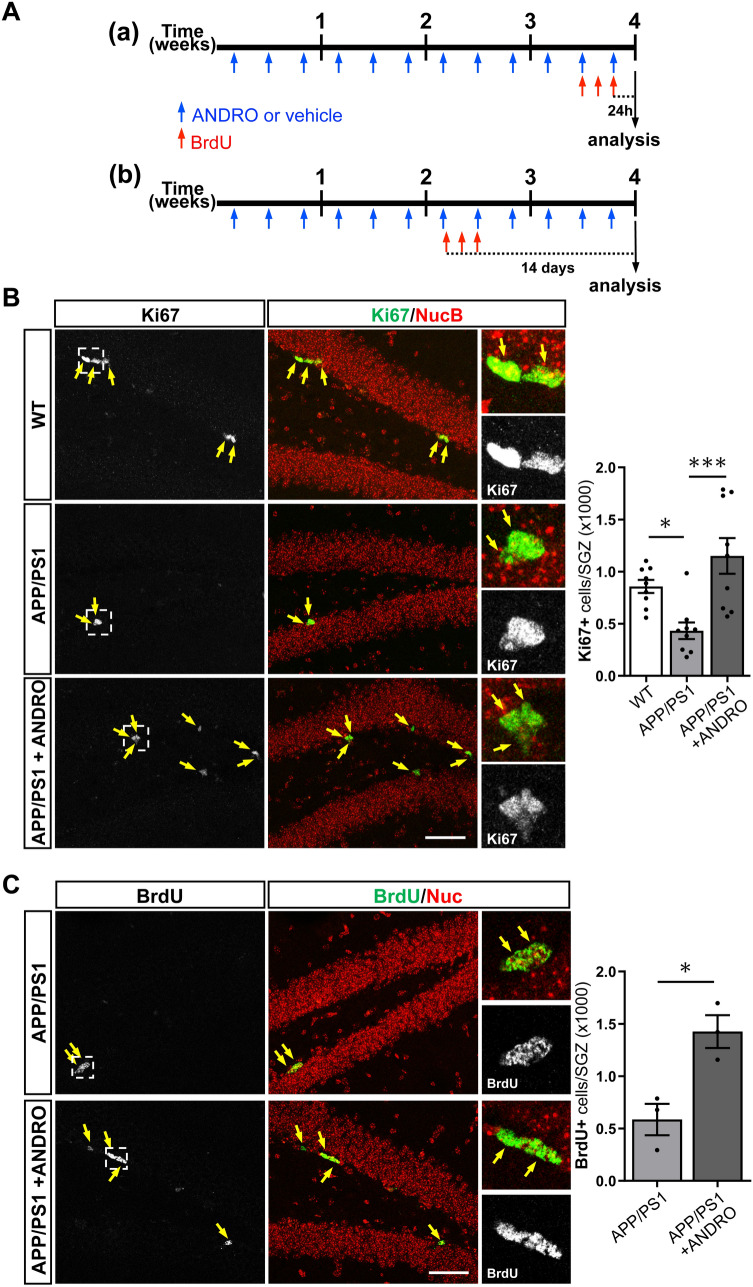
ANDRO increases cell proliferation in the dentate gyrus of APPswe/PSEN1ΔE9 mice. (**A**) Schematic representation of experimental procedure; 8-month-old APPswe/PSEN1ΔE9 mice were injected i.p. with 2 mg kg^−1^ ANDRO or vehicle solution 3 times a week for 4 weeks. Animals received a daily i.p. injection of 100 mg kg^−1^ BrdU the last 3 days of treatment and were sacrificed 24 h after the last BrdU injection (a) or received a daily i.p. injection of 100 mg kg^−1^ BrdU for 3 consecutive days and were sacrificed 14 days after the first BrdU injection (b). (**B**) Representative confocal images of the immunofluorescence of Ki67 in the dentate gyrus of control wild-type (WT), APPswe/PSEN1ΔE9 (APP/PS1) mice, and APP/PS1 mice treated with ANDRO. Images correspond to maximal projection of 10 µm z-stack. Scale Bar: 50 µm. Right panels show higher magnifications of single z-stack of dotted squares shown in left panels. Arrows indicate Ki67-positive (Ki67+) cells. The graph shows the quantification of the total number of Ki67+ cells in the subgranular zone (SGZ). Data are presented as mean ± SEM; WT = 9 mice; APP/PS1 = 9 mice; APP/PS1 + ANDRO = 9 mice. *p = 0.0445; ***p = 0.0005, one-way ANOVA followed by Bonferroni post-hoc test. (**C**) Representative confocal images of the immunofluorescence of BrdU in the dentate gyrus of APPswe/PSEN1ΔE9 mice injected with vehicle solution (APP/PS1) and APP/PS1 mice treated with ANDRO. Images correspond to maximal projection of 8 µm z-stack. Scale Bar: 50 µm. Right panels show higher magnifications of single z-stack of dotted squares shown in left panels. Arrows indicate BrdU-positive (BrdU+) cells. The graph shows the quantification of the total number of BrdU+ cells in the SGZ. Data are presented as mean ± SEM; N = 3 mice. * p = 0.0177, unpaired Student’s t-test.

Previously we determined that treatment with ANDRO increased the density of cells positive for the intrinsic proliferation marker Ki67 and for the immature neuronal marker doublecortin (DCX) in the dentate gyrus of APPswe/PSEN1ΔE9 mice^24^. Here, to further characterize the effect of ANDRO on neurogenesis we carried out two experimental designs (Fig. 1A): (a) To assess proliferation by nuclear incorporation of the nucleotide analog BrdU, 8-month-old APPswe/PSEN1ΔE9 mice were injected i.p. with 2 mg kg^−1^ ANDRO or vehicle solution 3 times a week for 4 weeks, the last 3 days of treatment animals received a daily i.p. injection of 100 mg kg^−1^ BrdU and were sacrificed 24 h after the last BrdU injection (Fig. 1A, a); (b) To evaluate neuronal differentiation of newborn cells, migration and development of newborn neurons, 8-month-old APPswe/PSEN1ΔE9 mice were injected i.p. with 2 mg kg^−1^ ANDRO or vehicle 3 times a week for 4 weeks, after 2 weeks of treatment the animals received a daily i.p. injection of 100 mg kg^−1^ BrdU for 3 consecutive days and were sacrificed 14 days after the first BrdU injection (Fig. 1A, b). In this experiment, age matched non-transgenic wild-type littermates receiving vehicle solution and BrdU were also included. In APPswe/PSEN1ΔE9 mice first pathological hallmarks of AD are detected at 4-month-old^34^, and impairments in neurogenesis are evident as early as 2 months of age^11^.

At the end of both experiments Ki67 staining was evaluated in the SGZ (Fig. 1B). A significant decrease in cells positive for Ki67 was observed in the dentate gyrus of APPswe/PSEN1ΔE9 mice compared to non-transgenic littermates (Fig. 1B), and as we previously reported^24^, an increase in Ki67-positive cells was observed in APPswe/PSEN1ΔE9 mice treated with ANDRO (Fig. 1B). As expected, an increase in Ki67-positive cells was also observed in wild-type mice treated with ANDRO (control = 793 ± 89; ANDRO = 1102 ± 99, p = 0,048). Proliferation was also evaluated by incorporation of BrdU (Fig. 1A, a). A significant increase in the total number of BrdU-positive (BrdU+) cells was observed in the SGZ of APPswe/PSEN1ΔE9 mice treated with ANDRO compared with control APPswe/PSEN1ΔE9 mice injected with vehicle solution (Fig. 1C). This result strongly supports that ANDRO induces cell proliferation in the SGZ of APPswe/PSEN1ΔE9 mice.

### ANDRO increases neural precursor cells and immature neurons in the dentate gyrus of APPswe/PSEN1ΔE9 mice

**Figure 2:**
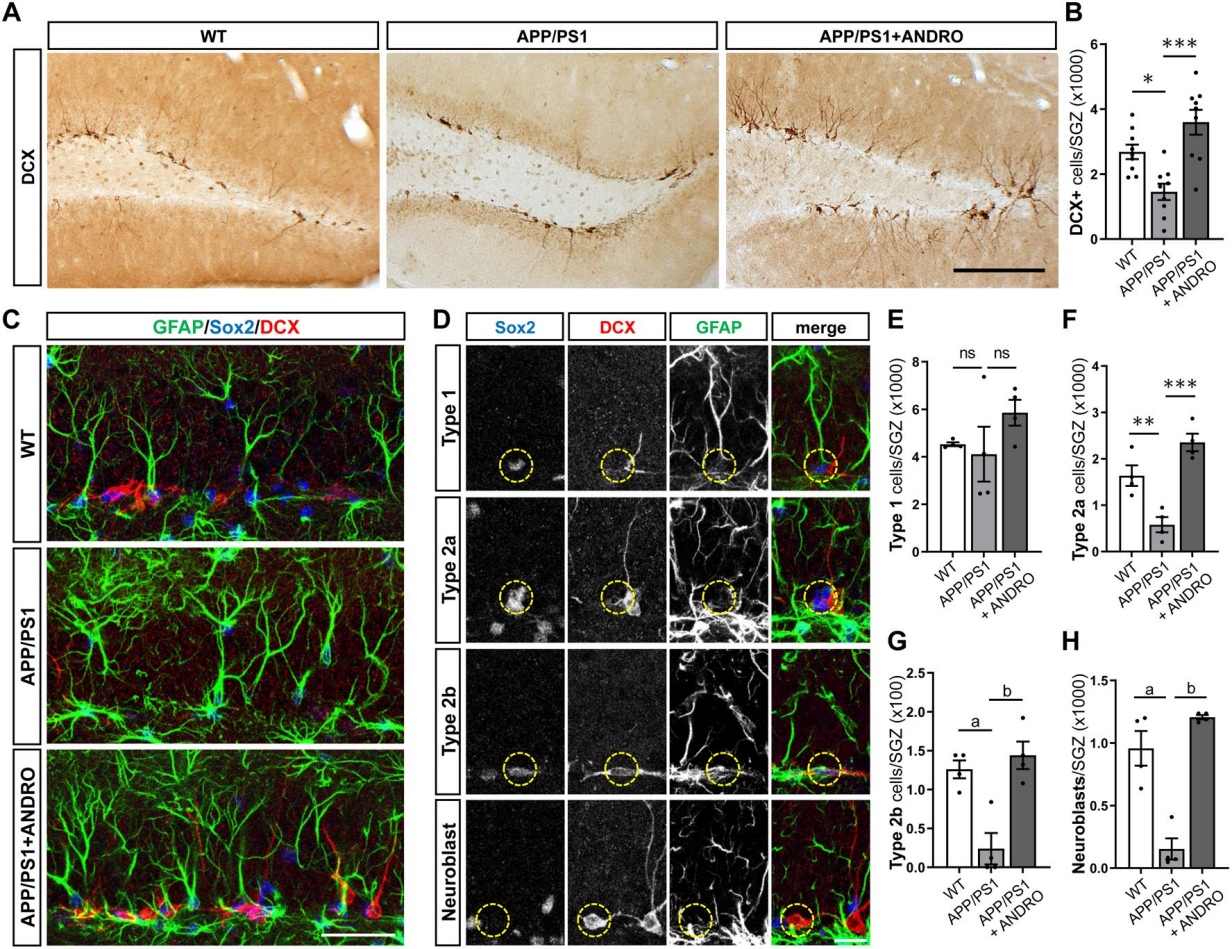
ANDRO increases neural precursor cells and immature neurons in the dentate gyrus of APPswe/PSEN1ΔE9 mice. (**A**) Representative immunostaining of immature neuronal marker DCX in the dentate gyrus of control wild-type (WT), APPswe/PSEN1ΔE9 (APP/PS1) mice, and APP/PS1 mice treated with ANDRO. Scale Bar: 200 µm. (**B**) Quantification of the total number of DCX+ cells in the granular cell layer (GCL). Data are presented as mean ± SEM; WT = 9 mice; APP/PS1 = 9 mice; APP/PS1 + ANDRO = 9 mice. *p = 0.0204, ***p < 0.0001, one-way ANOVA followed by Bonferroni post-hoc test. (**C**) Immunostaining of GFAP, Sox2 and DCX in WT, APP/PS1 mice, and APP/PS1 mice treated with ANDRO. Scale Bar: 40 µm. (**D**) Representative immunostaining of type 1, type 2a, type 2b cells and neuroblasts in the dentate gyrus identified by immunostaining with GFAP, Sox2 and DCX, and by cell morphology. Scale Bar: 20 µm. (**E–H**) Quantification of the total number of type 1 (**E**), type 2a (**F**), type 2b cells (**G**) and neuroblasts (**H**) in the SGZ of WT, APP/PS1, and APP/PS1 mice treated with ANDRO. Data are presented as mean ± SEM; WT = 4 animals; APP/PS1 = 4 animals; APP/PS1 + ANDRO = 4 animals. ns, non-significantly different. In (**F**), **p = 0.0098; ***p = 0.003. In (**G**), ^a^p = 0.0062; ^b^p = 0.0021. In (**H**), ^a^p = 0.0006; ^b^p < 0.0001; one-way ANOVA followed by Bonferroni post-hoc test.

To evaluate the effect of ANDRO on neurogenesis we first evaluated cells positive for DCX (Fig. 2A), a microtubule-associated protein that is transiently expressed during maturation of newborn neurons^35^. Reduced number of DCX-positive (DCX+) cells was observed in the dentate gyrus of APPswe/PSEN1ΔE9 mice compared to age-matched wild-type mice, and a significant increase was observed in APPswe/PSEN1ΔE9 treated with ANDRO (Fig. 2B).

In addition, we evaluated the neural stem/progenitor cells and neuroblasts population (Fig. 2C). These cells were identified by immunostaining with GFAP, Sox2 and DCX, and by cell morphology^36,37^. NSCs, also known as type 1 cells, are positive for GFAP and Sox2 labeling and extend a radial process into the granule cell layer (GCL) (Fig. 2D). These cells divide to generate type 2 neural progenitors that are rounded cells located in the SGZ that do not express GFAP and that are classified as type 2a or type 2b cells based on their neuronal commitment^38^. Type 2a cells are positive for Sox2 but not for DCX labeling, while type 2b cells are positive for both, Sox2 and DCX (Fig. 2D). Neuroblasts are positive for DCX but negative for GFAP and Sox2 labeling (Fig. 2D), are located in the SGZ and may exhibit small horizontal processes^35^. No significant changes were observed in the number of type 1 cells between wild-type, APPswe/PSEN1ΔE9 mice and APPswe/PSEN1ΔE9 mice treated with ANDRO (Fig. 2E). A significant decrease was observed in type 2a, type 2b and neuroblasts in APPswe/PSEN1ΔE9 mice compared to wild-type mice (Fig. 2F–H), and the number of these cells was significantly increased by ANDRO treatment (Fig. 2F–H).

Altogether, these results indicate that although there are no differences in the NSC population, there is a reduced number of neural progenitors and immature neurons in the dentate gyrus of APPswe/PSEN1ΔE9 mice compared to age-matched wild-type mice, and that these cells are increased in transgenic mice by the treatment with ANDRO.

### ANDRO does not affect neuronal differentiation in the dentate gyrus of APPswe/PSEN1ΔE9 mice

**Figure 3:**
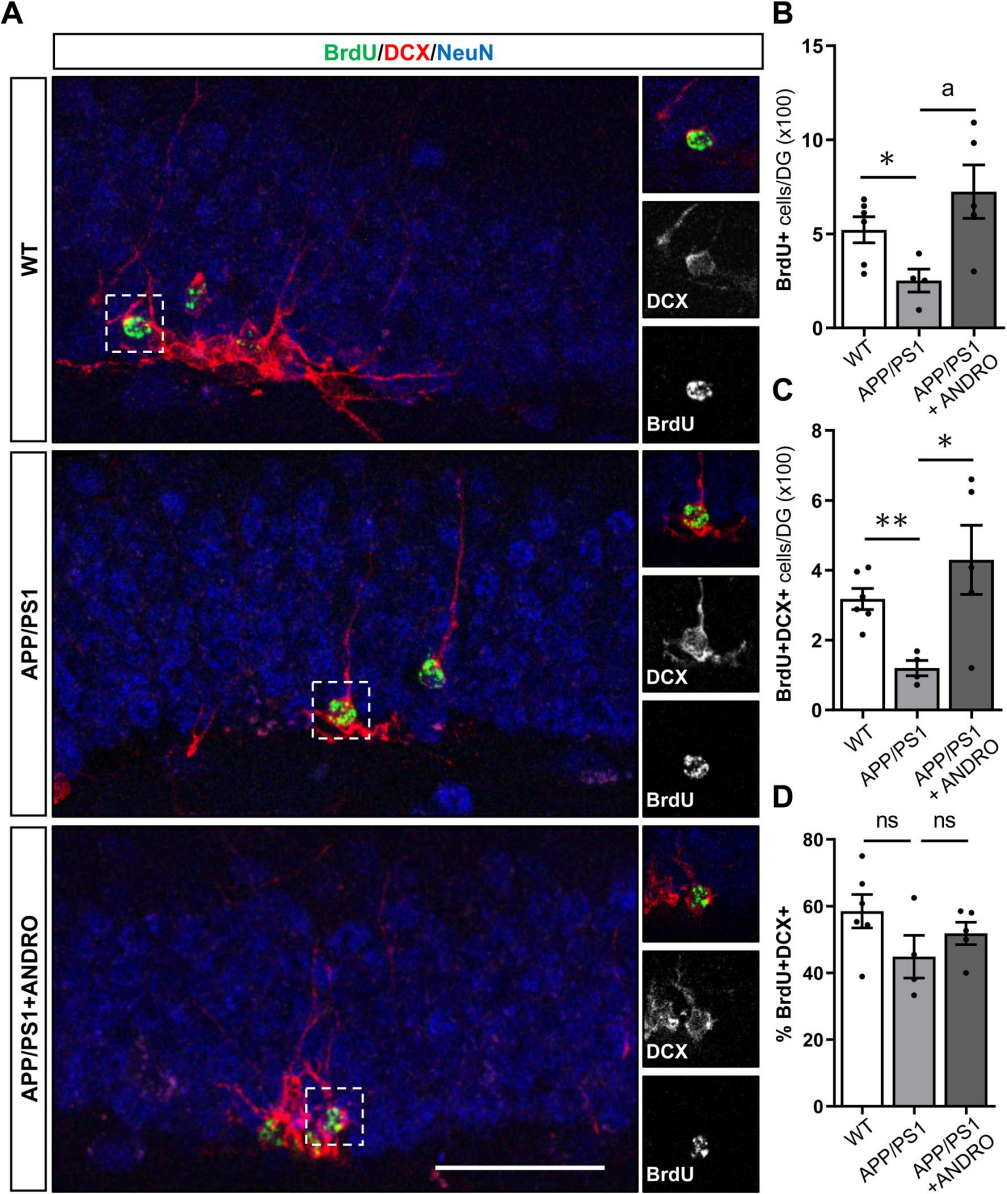
ANDRO does not affect neuronal differentiation in the dentate gyrus of APPswe/PSEN1ΔE9 mice. (**A**) Representative immunofluorescence staining of BrdU, and the neuronal markers DCX and NeuN in control wild-type (WT), APPswe/PSEN1ΔE9 (APP/PS1) mice, and APP/PS1 mice treated with ANDRO. Images correspond to maximal projection of 8 µm z-stack. Right panels show higher magnifications of single z-stack of dotted squares shown in left panels showing BrdU+DCX+ cells. Scale Bar: 50 µm. (**B**–**D**) Quantification of the total number of BrdU+ (**B**) and BrdU+DCX+ (**C**) cells in the GCL, and the percentage of BrdU+ cells expressing DCX (%BrdU+DCX+) (**D**), in animals treated with vehicle or ANDRO. Data are presented as mean ± SEM; WT = 6 mice; APP/PS1 = 4 mice; APP/PS1 + ANDRO = 5 mice. *p = 0.0255, ^a^p = 0.0272 (**B**); *p = 0.0298, **p = 0.0014 (**C**); ns, non-significantly different, unpaired Student’s t-test.

The increase in DCX+ cells in the dentate gyrus of APPswe/PSEN1ΔE9 mice treated with ANDRO might be mediated by the increased proliferation of neural precursor cells, and also by an increased differentiation of newborn cells into neurons. To assess neuronal differentiation of newborn cells DCX staining was evaluated in BrdU+ cells (Fig. 3A) 14 days after BrdU injection (Fig. 1A, b). Reduced number of BrdU+ cells was determined in the dentate gyrus of APPswe/PSEN1ΔE9 compared with wild-type mice, and in agreement with the proliferation analysis (Fig. 1C), a significant increase in total number of BrdU+ cells was observed in APPswe/PSEN1ΔE9 treated with ANDRO (Fig. 3B). Similar results were observed for total number of cells positive for BrdU and DCX (BrdU+DCX+, Fig. 3C), supporting that neurogenesis is reduced in APPswe/PSEN1ΔE9 compared to wild-type mice and that ANDRO stimulates this process in AD mice.

When assessing the percentage of BrdU+ cells expressing DCX, no differences were observed between wild-type mice, APPswe/PSEN1ΔE9 mice and APPswe/PSEN1ΔE9 mice treated with ANDRO (Fig. 3D), suggesting that ANDRO had no effect on neuronal differentiation.

These results suggest that the positive effect of ANDRO on neurogenesis in APPswe/PSEN1ΔE9 mice is not mediated by increased differentiation of neural precursor cells.

### ANDRO does not affect early morphological development or migration of newborn neurons

**Figure 4:**
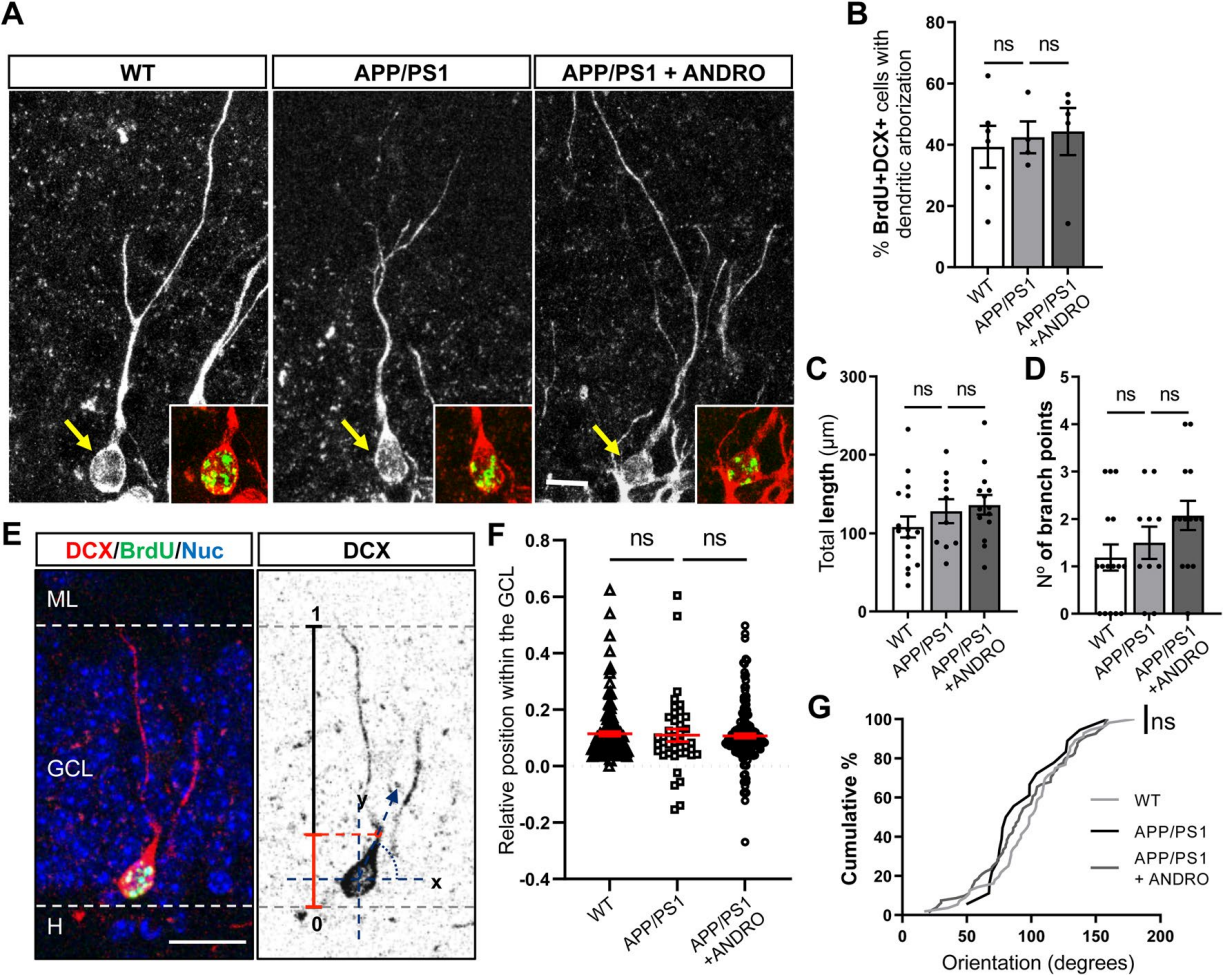
ANDRO does not affect early morphological development of newborn neurons. (**A**) Representative confocal images of BrdU+DCX+ cells showing dendritic arborization into the GCL towards the molecular layer in control wild-type (WT), APPswe/PSEN1ΔE9 (APP/PS1) mice, and APP/PS1 mice treated with ANDRO. Images correspond to maximal projection of 10 µm z-stack. Insets show z-stack of BrdU+DCX+ staining in cells indicated with the arrows (DCX: red; BrdU: green). Scale Bar: 10 µm (**B**) Quantification of the percentage of BrdU+DCX+ cells extending dendrites toward the molecular layer in WT, APP/PS1 mice and APP/PS1 mice treated with ANDRO. Data are presented as mean ± SEM; WT = 6 mice; APP/PS1 = 4 mice; APP/PS1 + ANDRO = 5 mice. ns, non-significantly different; one-way ANOVA test followed by Bonferroni post-hoc test. (**C**,**D**) Quantification of the total dendritic length (**C**) and number of branch points (**D**) of BrdU+DCX+ cells in WT, APP/PS1 and APP/PS1 mice treated with ANDRO. Data are presented as mean ± SEM; WT = 16 cells (N = 3 mice); APP/PS1 = 10 cells (N = 3 mice); APP/PS1 + ANDRO = 14 cells (N = 3 mice). One-way ANOVA test followed by Bonferroni post-hoc test. ns, non-significant. (**E**) Representative immunofluorescence of BrdU+DCX+ cell (left panel) and inverted DCX immunostaining (right panel) showing the schematic model of the analysis to assess the relative position of BrdU+DCX+ cells within the GCL, and the angular orientation of the initiation site (red dot), with the x-axis parallel to the GCL (0°–180°), the y axis pointing toward the hilus or ML (90° and 270°), and the origin at the center of the soma. (**F**) Quantification of the relative position of BrdU+DCX+ cells within the GCL. ns, non-significant, Mann–Whitney test. (**G**) Cumulative distribution plots of the angular orientation of the initiation site of BrdU+DCX+ cells. ns, non-significant, Kolmogorov–Smirnov test. (**D**,**E**): WT = 6 animals; APP/PS1 = 4 animals; APP/PS1 + ANDRO = 5 animals, N > 40 cells.

To assess whether ANDRO modulates the development of newborn neurons, we evaluated the dendritic arborization in BrdU+DCX+ cells (Fig. 4A). This analysis was carried out in cells positive for BrdU to compare immature neurons of the same age (Fig. 1A, b), some of which extended dendrites into the GCL towards the molecular layer (Fig. 4A) or showed tangential processes in the SGZ. No changes in the percentage of BrdU+ DCX+ extending dendrites into the GCL were observed between the three experimental groups (Fig. 4B). Of these branched neurons, no changes were observed in total dendritic length (Fig. 4C) or in the number of branch points (Fig. 4D).

We evaluated the migration of BrdU+DCX+ cells into the GCL (Fig. 4E). Although in APPswe/PSEN1ΔE9 mice more cells were observed in the hilus (relative position < 0), no significant differences were observed between wild-type and APPswe/PSEN1ΔE9 mice, or between APPswe/PSEN1ΔE9 mice treated with ANDRO or vehicle (Fig. 4F), being most of the cells located in the inner third of the GCL as previously reported for adult-born neurons^39^. Finally, the orientation of the primary dendrite of BrdU+DCX+ cells showing arborization was evaluated (Fig. 4E). No changes were observed in the orientation between the three experimental groups (Fig. 4G).

Altogether, the data suggest that treatment with ANDRO did not induce global changes in early morphological development of adult-born neurons.

### ANDRO improves spatial memory in APPswe/PSEN1ΔE9 mice

**Figure 5:**
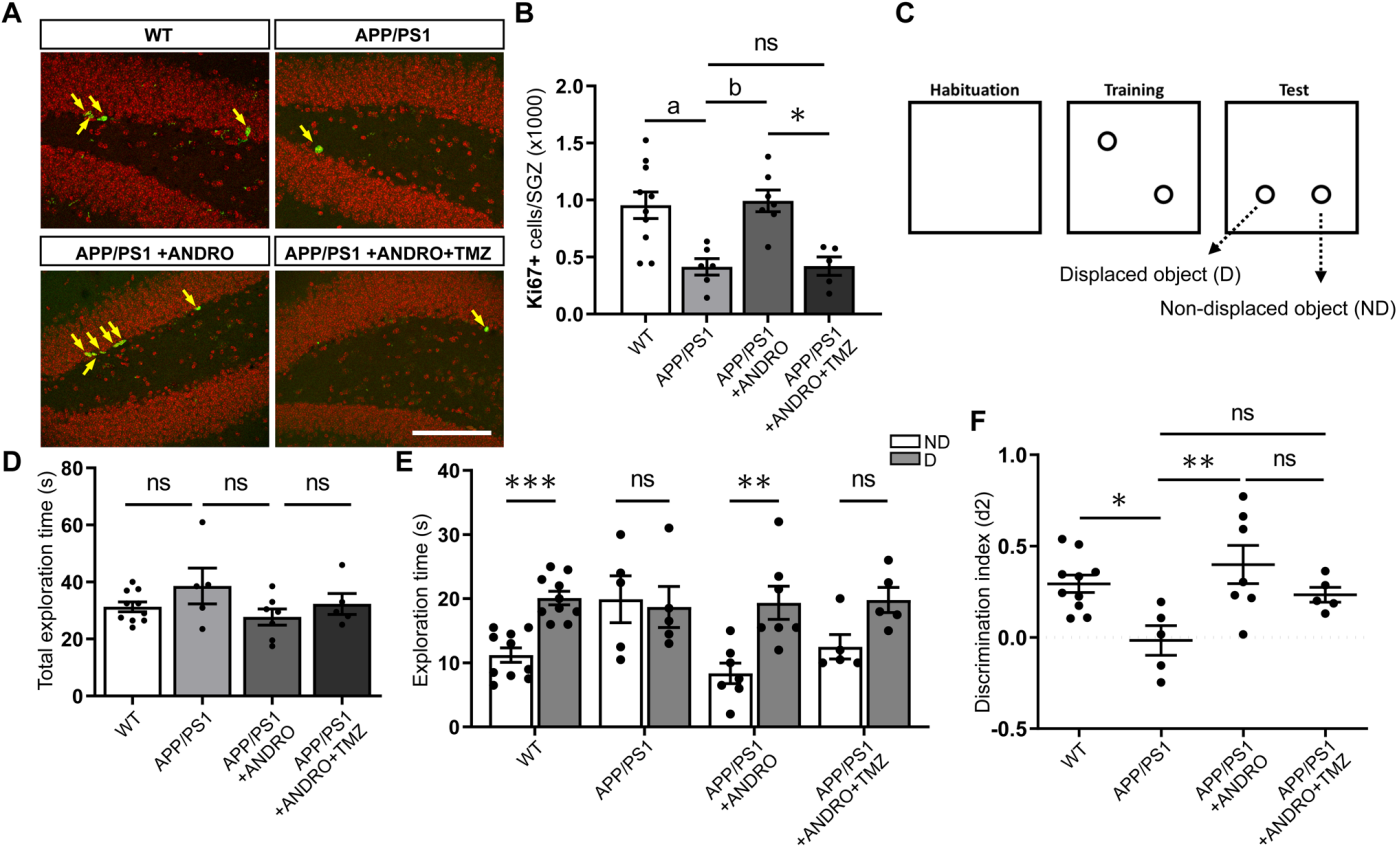
ANDRO improves spatial memory in APPswe/PSEN1ΔE9 mice. (**A**) Representative confocal images of the immunofluorescence of Ki67-positive (Ki67+, arrows) cells in the dentate gyrus of control wild-type (WT) mice, APPswe/PSEN1ΔE9 (APP/PS1) mice injected with vehicle, ANDRO or ANDRO + TMZ. Images correspond to maximal projection of 10 µm z-stack. Scale Bar: 50 µm. (**B**) Quantification of the total number of Ki67+ cells in the SGZ. One-way ANOVA test followed by Bonferroni post-hoc test. ^a^p = 0.0185; ^b^p = 0.0177; *p = 0.0139. ns, non-significant. (**C**) Schematic representation of the object location memory (OLM) test. (**D**) Quantification of the total exploration time in WT mice, APP/PS1 mice injected with vehicle, ANDRO or ANDRO + TMZ. ns, non-significant, One-way ANOVA test followed by Bonferroni post-hoc test. (**E**) Quantification of the time spent exploring the non-displaced (ND) and displaced (D) objects. ***p = 0.0002, **p = 0.0012, ns, non-significant. Mann–Whitney test. (**F**) Discrimination index (d2) calculated by dividing the difference of time spent to explore D and ND objects by the total time exploring both objects. *p = 0.0335, **p = 0.0067; ns, non-significantly different, one-way ANOVA test followed by Bonferroni post-hoc test. In (**B**–**F**), WT = 10 animals; APP/PS1 = 5 animals; APP/PS1 + ANDRO = 7 animals; APP/PS1 + ANDRO + TMZ = 5 animals.

To evaluate the effect of ANDRO on spatial memory we used the OLM task, a hippocampal-dependent task that involves exploiting the innate tendency of mice to explore novel items, and is inherently not stressful^40^. For this experiment, 8-month-old APPswe/PSEN1ΔE9 mice were injected i.p. with 2 mg kg^−1^ ANDRO or vehicle solution 3 times a week for 4 weeks. Also, a group of 8-month-old non-transgenic wild-type littermates mice received vehicle injections 3 times a week for 4 weeks. To assess the potential role of newborn cells in the cognitive effect of ANDRO, a group of APPswe/PSEN1ΔE9 animals were injected with ANDRO plus the DNA-alkylating agent TMZ (25 mg kg^−1^) that arrest cell proliferation and induces apoptosis of proliferating cells^41,42^. TMZ is able to cross the blood brain barrier^43,44^, and inhibits proliferation of NPCs in the hippocampus^45^. TMZ was administered together with ANDRO aiming to prevent the proliferative effect of ANDRO on APPswe/PSEN1ΔE9 mice (Fig. 5A,B).

The OLM task consisted of a context habituation day (empty training arena), a training day with two objects, and a testing day in which one object was moved to a novel location (displaced object, D) while the other object was not moved (non-displaced object, ND) (Fig. 5C). No significant differences were observed between the four experimental groups in total exploration time (time spent exploring both objects) during the 5 min testing session (Fig. 5D). When evaluating exploration time per object, wild-type mice preferentially explored the displaced object compared to the non-displaced object, suggesting that animals remembered the original training experience (Fig. 5E). On the other hand, APPswe/PSEN1ΔE9 mice similarly explored both objects indicating no preference for the displaced object, in agreement with previous studies demonstrating a spatial memory deficit in this mouse model of AD^46–48^. APPswe/PSEN1ΔE9 mice treated with ANDRO spent significantly more time exploring the displaced object, suggesting an improved performance in the OLM task. Although in animals co-injected with ANDRO plus TMZ no statistical differences were observed between the time exploring the displaced versus the non-displaced object, these animals showed a preference for the displaced object (Fig. 5E). When evaluating the discrimination index, statistical differences were observed between wild-type and APPswe/PSEN1ΔE9 mice, which showed equal preference for the two objects (Fig. 5F). A significant increase was observed for APPswe/PSEN1ΔE9 mice treated with ANDRO compared to control APPswe/PSEN1ΔE9 mice. Although mice treated with ANDRO plus TMZ showed a positive value for the discrimination index, this was not statistically different from the discrimination index of control APPswe/PSEN1ΔE9, or APPswe/PSEN1ΔE9 + ANDRO mice (Fig. 5F). This suggest that although mice treated with ANDRO + TMZ show preference for the displaced object, TMZ tends to reduce the effect of ANDRO.

## Discussion

Previously we reported that ANDRO treatment for 4 weeks increased the number of cells positive for the mitotic marker Ki67, which was also demonstrated in the present study, and increased DCX staining in the hippocampus of the APPswe/PSEN1ΔE9 mouse model of AD^24^. This animal model shows reduced hippocampal neurogenesis^7,11^, which was also observed in the present study when comparing newborn DCX+ cells in APPswe/PSEN1ΔE9 versus age-matched wild-type mice. Here we deepen in the effect of ANDRO on neurogenesis in APPswe/PSEN1ΔE9 mice by evaluating proliferation, neural precursor cells population, neuronal differentiation, newborn neurons development, and spatial memory.

First, by using the thymidine analog BrdU that is incorporated in the DNA of proliferating cells during DNA synthesis, we determined that ANDRO induced the number of proliferative cells in the dentate gyrus of APPswe/PSEN1ΔE9 mice. Therefore, by both strategies used, Ki67 staining and BrdU incorporation, the data strongly suggest that ANDRO induces cell proliferation in the hippocampus of APPswe/PSEN1ΔE9 mice. ANDRO treatment also increased the total number of type 2a/b neural progenitors, neuroblasts and newborn DCX+ cells in the dentate gyrus of AD mice, cell populations that were decreased in APPswe/PSEN1ΔE9 versus wild-type mice. Interestingly, no changes in type 1 cells were observed between the experimental groups, suggesting that neurogenesis impairment in AD mice and stimulation with ANDRO are not mediated by global changes in the NSC population. Additionally, ANDRO induced no changes in neuronal differentiation of newborn BrdU+ cells, suggesting that the observed increase in DCX+ cells is due to the effect in proliferation of neural precursor cells and not due to an increase in their differentiation. Intriguingly, we did not observe changes between wild-type and APPswe/PSEN1ΔE9 mice in the percentage of BrdU+ cells expressing DCX two-weeks after birth, suggesting that differentiation is not impaired in AD mice. However, we previously determined a reduced differentiation of newborn BrdU cells 24 h after the BrdU injection in APPswe/PSEN1ΔE9 mice^7^, and other studies have also suggested impaired differentiation in AD^11,49^. One possibility for this discrepancy is that there might be an increased elimination of cells unable to undergo proper differentiation and maturation in APPswe/PSEN1ΔE9 mice, a process that normally occurs in the adult hippocampus^50^. If so, the percentage of newborn cells positive for DCX may be increased after 2 weeks because of the elimination of undifferentiated cells, which might result in a strong decrease in total density of newborn DCX+ neurons even though the percentage of BrdU+ DCX+ cells appears unaffected. In agreement with this idea, it has been shown in different models of AD that survival of NPCs is affected^51^. Further analysis, including comparing the survival of newborn cells in wild-type versus APPswe/PSEN1ΔE9 mice, will be necessary to fully elucidate the contribution of abnormal neuronal differentiation and cell death to the strong differences in the total number of DCX + cells in APPswe/PSEN1ΔE9 versus wild-type mice.

One potential mechanism involved in the effect of ANDRO may include the inhibition of GSK-3β^52^, which in turn could stimulate neurogenesis through different mechanisms. GSK-3β inhibition might induce the Wnt/β-catenin signaling cascade. Accordingly, we previously determined that ANDRO treatment induced the activation of the Wnt/β-catenin signaling in the hippocampus of wild-type and APPswe/PSEN1ΔE9 mice^24,52^. Compelling evidence indicate that a downregulation of this signaling pathway is associated to the pathophysiology of AD^53–55^, and evidence suggest an association between Wnt signaling impairment and reduced neurogenesis in aging and AD^56^. The Wnt/β-catenin signaling pathway is a key signaling mechanisms regulating neurogenesis in the adult hippocampus^57–60^, e.g. it controls proliferation of NPCs through the expression of the Wnt target gene Cyclin D1^61^. Therefore, ANDRO might control proliferation through this Wnt-mediated mechanism. In addition, activation of Wnt signaling by different strategies was shown to reduce spatial memory impairment in APPswe/PSEN1ΔE9 mice^48,62^, which suggests that cognitive improvements induced by ANDRO in our and other studies^30–33^ might involve the activation of the Wnt signaling pathway.

In addition, inhibition of GSK-3β may have an impact on APP processing. GSK-3β plays a critical role in the development of the histopathological hallmarks of AD^63^, including downregulation of non-amyloidogenic cleavage of APP^64^. Furthermore, GSK-3β inhibition reduces the putative β-secretase BACE1-mediated cleavage of APP^65^. Also, it has been suggested that inhibition of Wnt signaling promotes the amyloidogenic proteolytic processing of APP, while Wnt signaling activation promotes the non-amyloidogenic processing of APP and reduces the levels of Aβ42 and its aggregates^66^. In accordance, ANDRO have shown to reduce Aβ levels and Aβ aggregates in animal models of AD^31,67^. In addition, in APPswe/PSEN1ΔE9 mice ANDRO affects the maturation of amyloid plaques^31^. Since Aβ deposition is linked to abnormal hippocampal neurogenesis^51,68^, by inhibiting GSK-3β ANDRO may stimulate neurogenesis through reducing Aβ deposition.

ANDRO effects may also involve the activation of Akt signaling. ANDRO was shown to modulate this signaling in the hippocampus of a rat model of chronic cerebral hypoperfusion (CCH) characterized by increased neurodegeneration, neuroinflammation and apoptosis^25^, as well as in neuroblastoma SH-SY5Y cells^69^. In CCH model, ANDRO treatment reduced the levels of the tumor suppressor PTEN and increased the levels of Akt. Akt modulates processes such as cell growth, proliferation, and survival^70^. In addition, stimulation of PI3K/Akt signaling enhances neurogenesis^71^, while inhibition of this pathway prevents the induction of neurogenesis by physiological stimulation^72,73^ which was associated to a reduced survival of newly generated neurons^72^. Therefore, through Akt signaling ANDRO might induce the survival of newborn neurons, which is affected in AD^51^. In support of this notion, based on BrdU incorporation assays, an estimated ~ 57% reduction in the number of BrdU + cells was observed in APPswe/PSEN1ΔE9 mice 14 days after BrdU administration (252 ± 61.4 cells, Fig. 2D) compared to 3 days after BrdU administration (586 ± 149.3 cells, Fig. 1C), while in APPswe/PSEN1ΔE9 mice treated with ANDRO, the estimated reduction in BrdU + cells was ~ 49% (3 dpi: 1426 ± 155.9 cells; 14 dpi: 725 ± 142.1). Therefore, ANDRO may induce proliferation and survival of NPC and immature neurons. We observed no changes in early morphological development of immature neurons, including dendritic arborization (total dendritic length and number of branch points) and angular orientation of dendrites, neither in migration of newborn neurons into the GCL. However, further analyses are needed to evaluate if ANDRO may stimulate maturation, integration and survival of adult-born neurons.

ANDRO might also restore neurogenesis in APPswe/PSEN1ΔE9 mice through its anti-inflammatory capacity. Neuroinflammation is considered a major feature of AD and other neurodegenerative diseases, a process including microglia and astrocyte activation, proinflammatory cytokines production and reactive oxygen species^74–76^. Increased microglia activity has been correlated with a decline in NSCs proliferation and neuronal differentiation and survival^77–79^, while anti-inflammatory treatment restores neurogenesis in inflammatory conditions^78,80^. ANDRO has shown anti-inflammatory effects in different models of AD. In the rodent Octodon degus that has been proposed as a natural model for AD since it spontaneously develops neuropathological hallmarks of AD pathology^81–83^, ANDRO reduced astrogliosis, oxidative stress and pro-inflammatory cytokines^67^. Also, treatment with nanoparticles loaded with ANDRO reduced astrocyte activation in TgCRND8 mouse model of AD^84^. In addition, ANDRO reduced the production of proinflammatory cytokines in Aβ-stimulated microglial cells^85^, and reduced astrocyte activation and the expression of proinflammatory cytokines in a model of CCH^27^. Reports indicate that ANDRO ameliorate inflammation by inhibiting the activation of nuclear factor-κB (NF-κB)^26,85^, which was identified as a critical mediator of stress-induced neurogenesis impairment^86^. Interestingly, the enzyme GSK-3β has the capacity to positively regulate NF-κB^87^, while the Wnt/β-catenin signaling pathway inactivates the transcriptional activity of NF-κB^88,89^. Therefore, ANDRO may reduce inflammation and promote neurogenesis through a mechanism involving the inhibition of GSK-3β and consequent activation of Wnt signaling.

We determined that ANDRO improved the performance of APPswe/PSEN1ΔE9 mice in the OLM task, which have shown to be dependent on the hippocampus for encoding, consolidation and retrieval^40^. This result is in agreement with previous studies showing that ANDRO improved the performance of animal models of AD in different spatial memory tasks^30–33,84^. It has been shown that altered hippocampal neurogenesis contributes to the learning and memory deficits in AD^90^, and increasing neurogenesis promotes cognitive performance in AD mice^91^. Although, co-treatment with the antimitotic drug TMZ tends to reduce the effect of ANDRO, it did not significantly prevent cognitive improvement. Of note, although TMZ treatment reduced the number of Ki67-positive cells, the decrease of DCX-positive neurons was not statistically significant (Supplementary Fig. 1), likely due to the high variability observed in the number of DCX cells in animals treated with the drug. Still, these results suggests that other effects of ANDRO in addition to the increase in neurogenesis (e.g. decrease in Aβ burden^31^), might underlie the observed cognitive improvement. In this context, it was showed in 5xFAD mice that increasing neurogenesis through genetic or pharmacologic methods alone results in slight cognitive enhancement, and full rescue requires also increasing brain derived neurotrophic factor (BDNF) levels^92^. Importantly, ANDRO has shown to induce BDNF levels in the hippocampus^27^, which may also contribute to the cognitive improvements of ANDRO in APPswe/PSEN1ΔE9 mice.

In summary, our results indicate that ANDRO stimulates neurogenesis in the hippocampus of APPswe/PSEN1ΔE9 by inducing proliferation of neural precursor cells. Although inhibition of proliferation tends to decrease the improvement in spatial memory performance induced by ANDRO, further analysis with genetic manipulation of neurogenesis in animals treated with ANDRO will provide more substantial evidence to support the contribution of newborn neurons to the cognitive effects. Altogether, our findings support that ANDRO is a natural product with potential therapeutic benefits for the treatment of brain conditions affecting neurogenesis and cognition.

## Materials and methods

### Animals and treatments

All experimental procedures involving mice were conducted in accordance with NIH and ARRIVE guidelines and were approved by the Bioethical Committee of Universidad Andrés Bello. Female APPswe/PSEN1ΔE9 (stock #004462, The Jackson Laboratory) and non-transgenic wild-type littermates were used for the experiments. Mice had access to water and food ad libitum in a 12:12 h light/dark cycle. At 8 month of age mice were injected i.p. with 2 mg kg^−1^ ANDRO (Sigma-Aldrich) or vehicle (DMSO, Sigma-Aldrich), 3 times a week for 4 weeks. Some animals received a daily i.p. injection of 100 mg kg^−1^ 5-Bromo-2′-deoxyuridine (BrdU, Sigma-Aldrich) for the last 3 days of ANDRO/vehicle treatment and were sacrificed 24 h after the last BrdU injection. Other group receive daily i.p. injection of 100 mg kg^−1^ BrdU for 3 consecutive days at 2 weeks of ANDRO/vehicle treatment and were sacrificed 2 weeks later. Eight-month-old mice used for behavioral testing were injected i.p. with 2 mg kg^−1^ ANDRO or vehicle 3 times a week for 4 weeks. Some animals were co-injected i.p. with 2 mg kg^−1^ ANDRO plus 25 mg kg^−1^ temozolomide (TMZ, AK Scientific, Inc) 3 times a week for 4 weeks.

### Perfusion, postfixation and tissue sectioning

Animals were anesthetized with a mixture of ketamine/xylazine (200 mg/kg, 20 mg/kg), and transcardially perfused with 50 ml ice-cold 0.9% NaCl, followed by 50 ml ice-cold 4% paraformaldehyde (PFA, Sigma-Aldrich/Merck Group) in 0.115 M NaH_2_PO_4_. Brains were removed and post-fixed in 4% PFA in PBS for 24 h at room temperature and then dehydrated in 30% sucrose. Brains were sectioned on a cryostat (Leica Microsystems) and collected in ice-cold-PBS in multiwell dishes as previously described^7^. Tissue sections were sequentially collected in 12 sets of serial slices of 50 μm. Each set contained 5–7 tissue sections covering the entire length of the hippocampus.

### Immunohistochemistry

Free-floating sections were washed three times in 0.15% Triton X-100 in PBS, and then incubated with 0.3% H_2_O_2_ for 30 min at room temperature. After washing three times in 0.15% Triton X-100 in PBS for 5 min, samples were incubated for 1 h with blocking solution (PBS, 5% BSA, 0.15% Triton X-100), and then incubated overnight at 4 °C with rabbit anti-DCX (4604S, Cell Signaling Technology Inc Danvers) diluted 1:400 in blocking solution. After washing, sections were incubated with biotinylated donkey anti-rabbit secondary antibody (1:250, BA-1000, Vector laboratories) for 1 h, washed in 0.15% Triton X-100 in PBS, and then incubated with avidin–biotin–peroxidase complex (1:125, Vectastain ABC Elite kit, PK-6101, Vector laboratories) for 1 h. Finally, sections were incubated with 0.3% H_2_O_2_ and 0.5 mg ml^−1^ 3,3-diaminobenzidine (DAB, Sigma) in Tris–HCL 50 mM (ph 7.6). Slides were washed, dehydrated, and coverslipped using Entellan^®^ (Merck Millipore). For in vivo analysis images were acquire in MOTIC^®^ (BA310, light microscope). For the analysis of total number of DCX+ cells, all tissues of 1–2 sets of the serial slices (see tissue sectioning) were acquired for each animal. Total number of cells counted in one set were multiplied by the total number of sets to obtain an estimation of the total number of DCX+ cells in the whole dentate gyrus.

### Immunofluorescence

Immunodetection of proliferation, progenitor and neuronal markers, and BrdU was carried out as previously described^7,93^. Primary antibodies used were rabbit anti-DCX (1:750, Cell Signaling Technology Inc.), goat anti-DCX (1:250, Santa Cruz Biotechnology), rabbit anti-Ki67 (1:250, Abcam), mouse anti-NeuN (1:300, Millipore), mouse anti-GFAP (1:8000, Sigma), goat anti-Sox2 (1:2000, R&D Systems), rat anti-BrdU (1:300, Abcam). As secondary antibodies Alexa (1:500, Molecular Probes) and DyLight (1:500, Abcam) conjugated antibodies were used. NucBlue (Life Technologies) was used as nuclear dye. Slices were mounted on gelatin-coated slides with Fluoromont-G (Electron Microscopy Sciences).

### Proliferation, differentiation, and morphological analyses

For proliferation, BrdU and Ki67-positive cells were counted in 1–2 sets of tissue sections (see tissue sectioning) using a fluorescence microscope (Nikon Eclipse Ti), and the number of cells was multiplied by the total number of tissues sets. For the analyses of neural precursor cells and neuroblasts, differentiation and migration, images were acquired by confocal laser microscopy (Leica TCS SP8 microscope), with a 40X objective and a z-axis interval of 1 µm. z-projections were made with ImageJ software (NIH). To analyze neural precursor cells and neuroblasts, all tissues in one set of tissue sections were analyzed for GFAP, Sox2 and DCX staining, and the number of cells counted was multiplied by the total number of sets. For differentiation analysis, all BrdU-positive cells in one set of tissue sections were analyzed for DCX staining. For morphological analysis, BrdU+DCX+ cells were acquired with a 60X objective by confocal laser microscopy (Leica TCS SP8 microscope) using 40–50 z-axis interval of 0.5 μm. Analysis of total dendritic length, and number of branch points were made using the Simple Neurite tracer plugin using FIJI software^94^. Angular orientation and position of newborn neurons within the GCL was evaluated as described previously^95,96^ in all BrdU+DCX+ cells of one set of tissue sections.

### Object location memory (OLM) test

The OLM test was performed as previously described^97^ with some modifications^98^. Briefly, animals were habituated for 5 min in an apparatus that contained a cage inside an insonorized chamber. During the training session, the cage contained two identical objects placed in opposite quadrants of the chamber; animals were placed in the center of the cage and left to explore for 5 min. In the test session, one object was moved to a different position (displaced object, D), while the other object was not moved (none-displaced, ND); animals were left to explore for 5 min. The exploration time was recorded and defined as time spent sniffing or touching the object with the nose and/or forepaws. Total exploration time indicates the time spent exploring both objects (D + ND). Animals that did not explore a total of 15 s for both objects during the testing session were excluded from the analysis. The discrimination index was calculated by dividing the difference of time spent to explore D and ND objects by the total time exploring both objects [discrimination index: (time D − time ND)/(time D + time ND)]. Timing and video analysis was conducted by an experimenter blind to the experimental conditions.

### Statistical analysis

All statistical analyses were performed using Prism Software 9 (GraphPad Software, LLC). All data were checked for normality using Shapiro–Wilk test. For data with normal distribution to compare mean values between two groups a two-tailed unpaired Student’s *t*-test was used with a significance level set to α = 0.05. For comparison between more than two groups, one-way ANOVA followed by Bonferroni multiple comparison post-hoc test was used to determine statistical significance. For data with non-normal distribution two-tailed Mann–Whitney test was used to compare mean values between two groups. For cumulative distribution of dendritic initiation site, the Kolmogorov–Smirnov (K–S) test was used. In all graphs the data represent the mean ± SEM. Number of animals per experimental group, or number of neurons analyzed in each experiment, is indicated in each Figure legend. p < 0.05 was considered statistically significant.

## Supplementary Information

Supplementary Figure 1.

## Data Availability

The datasets generated during and/or analyzed during the current study are available from the corresponding author on reasonable request.
