# Supplementary material for: Andrographolide promotes hippocampal neurogenesis and spatial memory in the APPswe/PS1ΔE9 mouse model of Alzheimer’s disease
Source: Sci Rep. 2021 Nov 25;11:22904. doi: 10.1038/s41598-021-01977-x (PMC8616902; doi:10.1038/s41598-021-01977-x)
Supplement: Supplementary file 1 — Supplementary Figure 1. [file 41598_2021_1977_MOESM1_ESM.pdf]

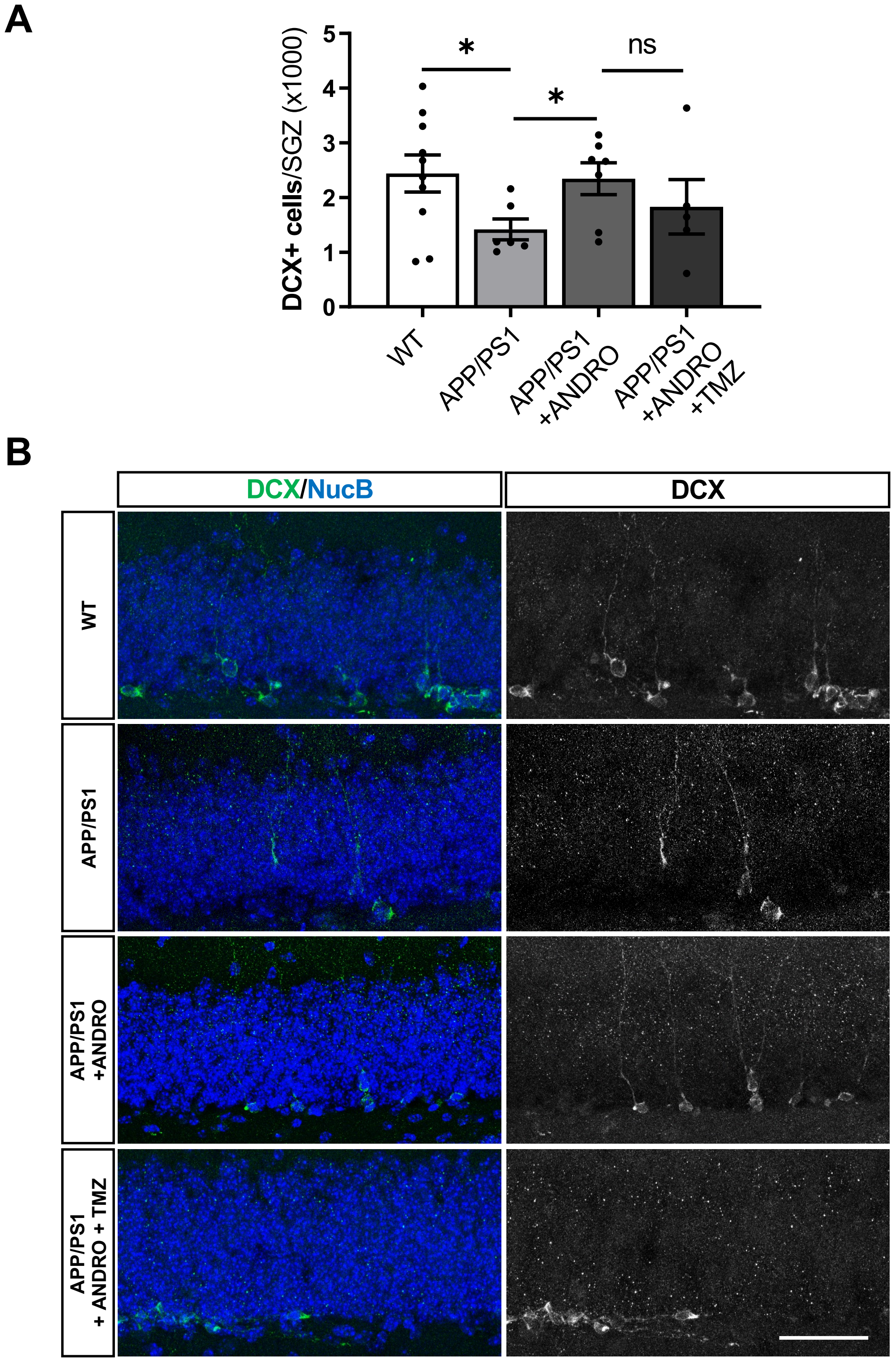

Supplementary Figure S1. Analysis of DCX-positive cells in animals subjected to OLM task. (A) Quantification of the total number of DCX+ cells in the granular cell layer (GCL) of all experimental groups subjected to OLM task. WT = 10 animals; APP/PS1 = 5 animals; APP/PS1+ANDRO = 7 animals; APP/PS1+ANDRO+TMZ = 5 animals. *$p<0.05$. ns, non-significant **(B)** Representative immunostaining of DCX in all experimental groups. Scale Bar: 50 μm.
